# Quantitative Chemical Proteomics Reveals Resveratrol Inhibition of A549 Cell Migration Through Binding Multiple Targets to Regulate Cytoskeletal Remodeling and Suppress EMT

**DOI:** 10.3389/fphar.2021.636213

**Published:** 2021-03-26

**Authors:** Xiao Chen, Yutong Wang, Jing Tian, Yurou Shao, Bo Zhu, Jigang Wang, Zichun Hua

**Affiliations:** ^1^ School of Medicine and Holistic Integrative Medicine and College of Pharmacy, Nanjing University of Chinese Medicine, Nanjing, China; ^2^ School of Biopharmacy, China Pharmaceutical University, Nanjing, China; ^3^ Artemisinin Research Center, and Institute of Chinese Materia Medica, China Academy of Chinese Medical Sciences, Beijing, China; ^4^ The State Key Laboratory of Pharmaceutical Biotechnology, School of Life Sciences, Nanjing University, Nanjing, China

**Keywords:** resveratrol, quantitative chemical proteomics, target identification, cytoskeletal remodeling, EMT

## Abstract

Resveratrol (RSV), a health-promoting natural product, has been shown to affect various cellular processes in tumor cells. However, the specific protein targets of RSV and the mechanism of action (MOA) of its anticancer effect remain elusive. In this study, the pharmacological activity of RSV was first evaluated in A549 cells, and the results showed that RSV significantly inhibited A549 cell migration but did not affect cell viability. To elucidate the underlying mechanism, a quantitative chemical proteomics approach was employed to identify the protein targets of RSV. A total of 38 target proteins were identified, and proteomic analysis showed that the targets were mainly involved in cytoskeletal remodeling and EMT, which were verified by subsequent *in vitro* and *in vivo* assays. In conclusion, RSV inhibits A549 cell migration by binding to multiple targets to regulate cytoskeletal remodeling and suppress EMT.

## Introduction

Lung cancer has become one of the critical causes of cancer death throughout the world due to the prevalent complication of apoptosis resistance against different anticancer agents (Ramezanpour et al., 2014). Because of the typical asymptomatic progression of lung cancer at an early stage, it is normally diagnosed at an advanced stage (Moghadamtousi et al., 2014), leading to a low 5-year survival rate for patients (Siegel et al., 2012). Furthermore, most survivors suffer from lung dysfunction in the long run, which causes a great burden to their families and society. However, traditional treatments, such as surgery and chemotherapy, exert limited therapeutic effects due to their side effects and low sensitivity. Therefore, improving overall survival among patients with lung cancer has become the goal of emerging treatments.

Recently, an accumulating amount of attention has been placed on the development of novel natural anticancer compounds capable of inhibiting multistep tumorigenesis with minimal adverse effects (Aggarwal et al., 2003; Signorelli and Ghidoni, 2005). Resveratrol, a polyphenol present in a variety of fruits and vegetables exhibiting low toxicity (Cottart et al., 2010; Crawford, 2014), exerts anticancer activity among various cancers, including colorectal cancer (Patel et al., 2010), breast cancer (Scarlatti et al., 2008), pancreatic cancer (Shankar et al., 2011), gastric cancer (Zulueta et al., 2015), and prostate cancer (Harper et al., 2007). Of interest, RSV has also been utilized to treat lung cancer (Bae et al., 2011; Zhang et al., 2013; Gu et al., 2015; Yuan et al., 2015). Reports have shown that RSV affects a large variety of cellular processes in lung cancer cells, including the cell cycle (Abe et al., 2010; Yuan et al., 2015), mitochondrial function (Gu et al., 2016), oxidative stress (Gu et al., 2015), and epithelial-to-mesenchymal transition (Wang et al., 2013), to inhibit cell proliferation and migration. These findings expand our understanding of the role of RSV in lung cancer cells, but the exact mechanism is not clear. Moreover, the specific target proteins of RSV in lung cancer cells remain elusive, also increasing the difficulty of mechanistic elucidation and new therapeutic target discovery (Chen et al., 2017b).

In recent years, quantitative chemical proteomics approaches have been widely applied to identify the protein targets of active small molecules to elucidate their mechanism of action and side effects (Cheng et al., 2014; Wang et al., 2014; Wang et al., 2016a). The approach utilizes a probe, which is structurally similar to its parent molecule and retains the pharmacological activity of interest, to interact with the protein targets in living cells or cell lysates (Chen et al., 2020). Then, the probe-protein complexes are enriched with affinity technology. Finally, the proteins are digested with trypsin, and the peptides are identified with mass spectrometry (Chen et al., 2017a). Therefore, the protein targets of small molecules are identified, and their MOA and side effects could be predicted with proteomic analysis tools.

In the present study, we first evaluated the pharmacological activities of RSV on A549 lung cancer cells. To elucidate the mechanism involved, we employed a quantitative chemical proteomics approach to identify the protein targets of RSV in A549 cells. Based on the proteomic data, the cellular processes and pathways modulated by RSV were predicted with MetaCore software and validated with subsequent *in vitro* and *in vivo* assays. Our study comprehensively identified the protein targets of RSV in an unbiased manner and elucidated its MOA in A549 cells, providing new therapeutic targets for lung cancer treatment.

## Materials and Methods

### Cell Culture

A549 and H226 cells were obtained from the American Type Culture Collection (ATCC, Philadelphia, PA, United States). The cells were maintained in tissue culture using F12K medium (A549, HyClone, Logan, UT, United States) or RPMI1640 medium (H226, HyClone, Logan, UT, United States) supplemented with 10% fetal calf serum (HyClone, Logan, UT, United States) and 1% penicillin-streptomycin (Invitrogen, Carlsbad, CA, United States). All cells were cultured in a humidified CO_2_ incubator at 37°C.

### CCK-8 Assay

5 × 10^3^ cells in 50 μL medium per well were seeded on 96 well plate and incubated at 37°C overnight, then treated with different concentration of RSV for different time span. 10 μL CCK-8 solution per well was added and incubated for 4 h at 37°C. Absorbance at 650 nm was measured.

### The Real-Time Cell Analyzer Assay

The RTCA system was applied to monitor cell migration and proliferation by using CIM-plates and E-plates. A549 or H226 cells were cultured in F12K or PRMI1640 with 10% fetal bovine serum for 24 h and followed by compound treatment. Normal culture medium or serum free medium was then placed in the lower chamber. The plate was left to settle for 30 min at room temperature in sterile conditions. The upper chamber was then mounted and 25 μL of serum free medium was added to each well and left to equilibrate in the incubator for 1 h at 37°C and 5% CO_2_. After the incubation, a background reading was taken for each well. A549 or H226 cells were prepared in serum free medium, 40,000 cells and compounds were plated into each well of the upper chamber of the CIM-plates or E-plates, and fresh medium was added to make up a total volume of 180 μL. Readings were recorded initially at 25 scans at 5 min intervals and then followed by scans at every 10 min intervals until the end of the experiment (up to 24 h). As an additional control, serum free medium was placed in the lower chamber and all other CI values were normalized to this baseline.

### Trans-Well Assay

A549 or H226 cells were seeded at a density of 5 × 10^4^ cells onto the inserts (8 mm pore size) in 24-well tissue culture plates. The cells were incubated in F12K or PRMI1640 medium supplemented with 2% serum and various concentrations of RSV. After 12 h of incubation, the cells on the upper side of the inserts were removed with a cotton swab. The cells that migrated to the lower side of the inserts were stained with 1% crystal violet and counted under a microscope.

### Cell Invasion Assay

A549 or H226 cells were seeded at a density of 3 × 10^5^ cells onto ECM gel-coated inserts (8 mm pore size) in 24-well tissue culture plates. The cells were incubated in F12K or PRMI1640 medium supplemented with 2% serum and various concentrations of RSV. After 18 h of incubation, the cells on the upper side of the inserts were removed with a cotton swab. The cells that migrated to the lower side of the inserts were stained with 1% crystal violet and counted under a microscope.

### Synthesis of Resveratrol Probe

HATU (800 mg, 2.1 mmol) was added into a solution of biotin (50 mg, 2.05 mmol) in DMF (20 ml) at 0°C. Then RSV (502 mg, 2.2 mmol) and DIPEA (323 mg, 2.5 mmol) were added under Ar atmosphere. The mixture was stirred for 3 h at room temperature. The mixture was concentrated in vacuo, the residue was purified by column chromatography with DCM: CH_3_OH = 4:1 to give the title compound as a white solid (110 mg, 11%). LCMS: calcd for C_24_H_26_N_2_O_5_S 455.1 (M + H)^+^, found 454.8.

### *In Vitro* Labeling of A549 Cells Lysate by RSV-P

A549 cells were grown in 150 mm dishes until 80–90% confluency was reached. The cells were washed twice with PBS after removal of the media. The cells were lysed with 20 mM Tris (pH 7.5), 150 mM NaCl and 1% Triton X-100. The cell lysate was centrifuged at 10,000 rpm for 45 min at 4°C to remove debris and insoluble fraction. Protein concentration of the lysates was determined with BCA protein quantitation kit. Equal amount of proteins (4 mg) were treated with streptavidin beads attached by RSV-P or DMSO for 4 h at room temperature to enrich the labeling proteins. The beads were washed with PBS (0.1% SDS), PBS and double distilled water several times to remove non-specific binding proteins.

### iTRAQ Labeling of the Tryptic Peptides

Labeling was performed using iTRAQ Reagent-8Plex reagent (SCIEX; Foster City, CA, United States). Two negative controls (DMSO) and two RSV-P pull-down samples were labeled with reagent 115, 116, 113 and 118 respectively. Briefly, after drying and reconstituting with 30 μL 0.5 M TEAB, the digested peptides were reacted with respective iTRAQ reagents for 3 h at room temperature. Afterwards, the labeled samples were pooled together and subjected to strong cation exchange and desalting. After desalting, the iTRAQ labeled peptide sample was dried and re-dissolved in 80 μL of 2% acetonitrile (ACN) containing 0.05% formic acid (FA).

### Mass Spectrometry for Proteins Identification

TripleTOF 5600 system (AB SCIEX, Foster City, CA, United States) was used to obtain mass-spectrometry spectrum in high resolution mode with more than 30,000 resolution (250 ms accumulation time per spectrum and a mass range of 400–1,250 m/z) and mass-spectrometry/mass-spectrometry spectrum in high sensitivity mode with more than 15,000 resolution. For each mass-spectrometry spectrum, a maximum of 20 precursors with a charge state between two and four were chosen for fragmentation. Also, the signals were accumulated for a minimum of 100 ms per spectrum and dynamic exclusion time was set at 15 s.

### Western Blotting

Total proteins were extracted from cells and separated using 10% SDS-PAGE and then electrophoretically transferred to a nitrocellulose membrane (Millipore; HATF00010). The membrane was blocked with 5% milk in PBST and incubated with primary antibody and secondary antibody. Target proteins were detected with ECL detection reagent (Thermo Scientific; 34075).

### Cytoskeleton Staining

A549 cells were grown in six-well plate at 37°C for 24 h and followed by RSV treatment. Wash cells twice with prewarmed phosphate-buffered saline (PBS). Fix the sample in 3.7% formaldehyde solution in PBS for 10 min at room temperature. Wash the cells with PBS for two or more times. Place each coverslip in a glass petri dish and extract it with a solution of acetone at ≤ −20°C or 0.1% Triton X-100 in PBS for 3–5 min. Wash the cells again. Dilute 5 µL methanolic stock solution of phallotoxins dye (Thermofisher, T7471) into 200 µL PBS for each cover-slip to be stained. To reduce nonspecific background staining with these conjugates, add 1% bovine serum albumin (BSA) to the staining solution. Place the staining solution on the coverslip for 20 min at room temperature (generally, any temperature between 4° and 37°C is suitable). To avoid evaporation, keep the coverslips inside a covered container during the incubation. Wash the sample again. Then use DAPI to stain nucleus.

### qRT-PCR Assay

Total RNA preparation and reverse transcription were performed as described previously (Mochizuki et al., 2006). PCR for EMT-related genes was carried out using the following primers: E-cadherin (forward 5′- CGA​GAG​CTA​CAC​GTT​CAC​GG-3′ and reverse 5′- GGG​TGT​CGA​GGG​AAA​AAT​AGG-3′); *β*-catenin (forward 5′- GCA​GGG​AGG​TGT​ACC​CGT​A -3′ and reverse 5′- GGG​GTT​TCC​TTA​GTC​AGG​ACA -3′); Fibronectin (forward 5′- GAG​TTG​TCG​TGG​TCC​CTC​AG′ and reverse 5′- TGG​AGG​CGG​CAT​CAT​AGT​TG -3′); Vimentin (forward 5′- GAC​GCC​ATC​AAC​ACC​GAG​TT -3′ and reverse 5′- CTT​TGT​CGT​TGG​TTA​GCT​GGT -3′); and N-cadherin (forward 5′- TCA​GGC​GTC​TGT​AGA​GGC​TT -3′ and reverse 5′- ATG​CAC​ATC​CTT​CGA​TAA​GAC​TG -3′).

### RhoA Activation Assay

Prior to lysis in RhoA G-LISA lysis buffer (Cytoskeleton, Denver, CO, United States), A549 cells were subjected to time course treatment with different concentrations of RSV as indicated. The RhoA GTPase assay was performed according to the manufactures protocol.

### Animal Experiments

To rule out the contribution of host immune response, a nude mouse model was used in this study. Female BALB/c mice aged 4–6 weeks with a body weight of 16–18 g was purchased from the Laboratory Animal Center of Yangzhou University (Yangzhou, China). The animals were housed under controlled conditions with a room temperature of 25 ± 2°C and 12 h/12 h light/dark cycle. All the animals were allowed to access to food and water in specific pathogen-free conditions.

All animals (*n* = 60) were injected with human A549 cells (5 × 10^6^/0.2 ml) in the right armpit. One week later, the tumor size was speculated to reach a mean diameter of 3–5 mm, and the animals were randomly divided into four groups (*n* = 10 for each group), which were intraperitoneally injected with 1% DMSO in PBS, low concentration of RSV (15 mg/kg), medium concentration of RSV (30 mg/kg) and high concentration of RSV (50 mg/kg) each other day respectively. The body weight and tumor size were measured each other day. All the mice were sacrificed when the tumors grew to about 1500 mm^3^. Genomes and proteins were extracted from the tumors for subsequent experiments.

### Statistical Analysis

GraphPad prism 5.0 was used for statistical analysis. Data was summarized as mean ± SEM. One-way ANOVA was used to determine the significant differences between groups. Results were considered to be significant for *p*-values of <0.05.

## Results

### Resveratrol Exhibits no Cytotoxicity in the A549 Cell Line but Inhibits Cell Migration and Invasion in a Dose-and Time-Dependent Manner

**FIGURE 1:**
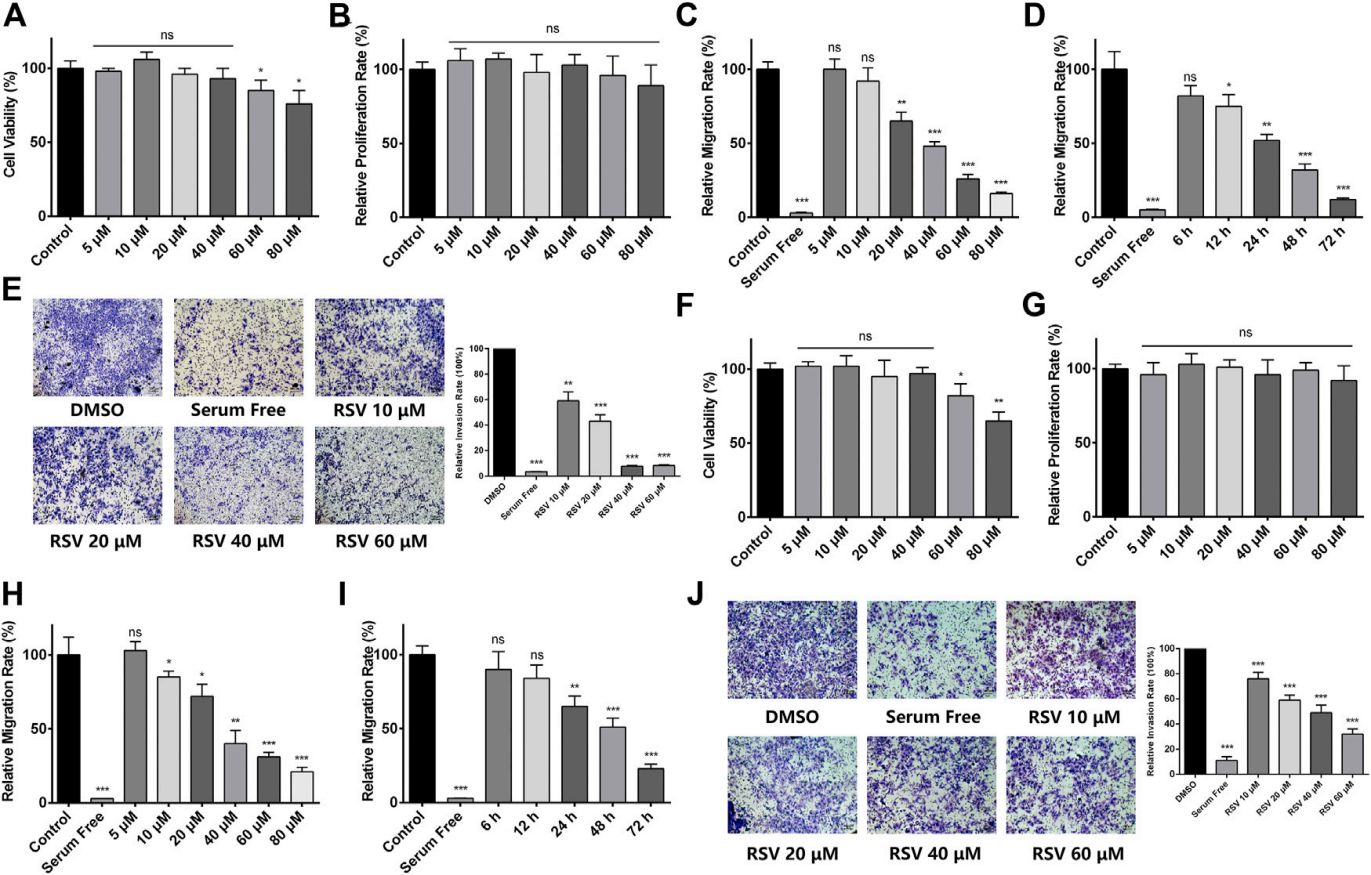
**(A)** Effect of different concentrations of RSV on A549 cell viability for 48 h; **(B)** Effect of different concentrations of RSV on A549 cell proliferation for 24 h **(C)** Effect of different concentrations of RSV on A549 cell migration for 24 h; **(D)** The migration rate of A549 cells treated with 38 μM RSV for different durations; **(E)** Effect of different concentrations of RSV on A549 cell invasion; **(F)** Effect of different concentrations of RSV on H226 cell viability; **(G)** Effect of different concentrations of RSV on H226 cell proliferation for 24 h; **(H)** Effect of different concentrations of RSV on H226 cell migration for 24 h; **(I)** The migration rate of H226 cells treated with 38 μM RSV for different durations; **(J)** Effect of different concentrations of RSV on H226 cell invasion. *p < 0.05, **p < 0.01, ***p < 0.005.

To evaluate the effect of RSV on A549 cell proliferation, a CCK-8 assay was performed. We treated A549 cells with different concentrations of RSV for 48h, and no obvious cytotoxicity of RSV was observed at concentrations ranging from 5 to 40μM, while RSV inhibited A549 cell viability at the concentration from 60 to 80μM (Figure 1A). Next, we applied a real-time cell analyzer (RTCA) to detect the effect of RSV on A549 cell proliferation and migration. Results showed that RSV has no effect on A549 cell proliferation (Figure 1B). Treatment with RSV for 24h significantly inhibited A549 cell migration in a concentration-dependent manner (Figure 1C), and the IC_50_ value (the concentration at which RSV treatment inhibits 50% of the migration rate of A549 cells) was 38μM, based on which we adopted 38μM as our experimental concentration in subsequent assays. The effect of different concentrations of RSV on A549 cell migration was also confirmed by trans-well assay (Supplementary Figure S1). In addition, we treated A549 cells with 38μM RSV for different durations from 0 to 72h, and RTCA results showed that RSV inhibited A549 cell migration in a time-dependent manner (Figure 1D). A trans-well assay was also performed to evaluate the effect of RSV on A549 cell invasion, and we found that RSV remarkably suppressed A549 cell invasion in a dose-dependent manner (Figure 1E). Another human lung cancer cell line, H226, was employed to validate the pharmacological effect of RSV on lung cancer cells, and similar results were observed (Figures 1F–J; Supplementary Figure S2).

Identification of RSV targets in A549 cells with a chemical quantitative proteomic approach.

**FIGURE 2:**
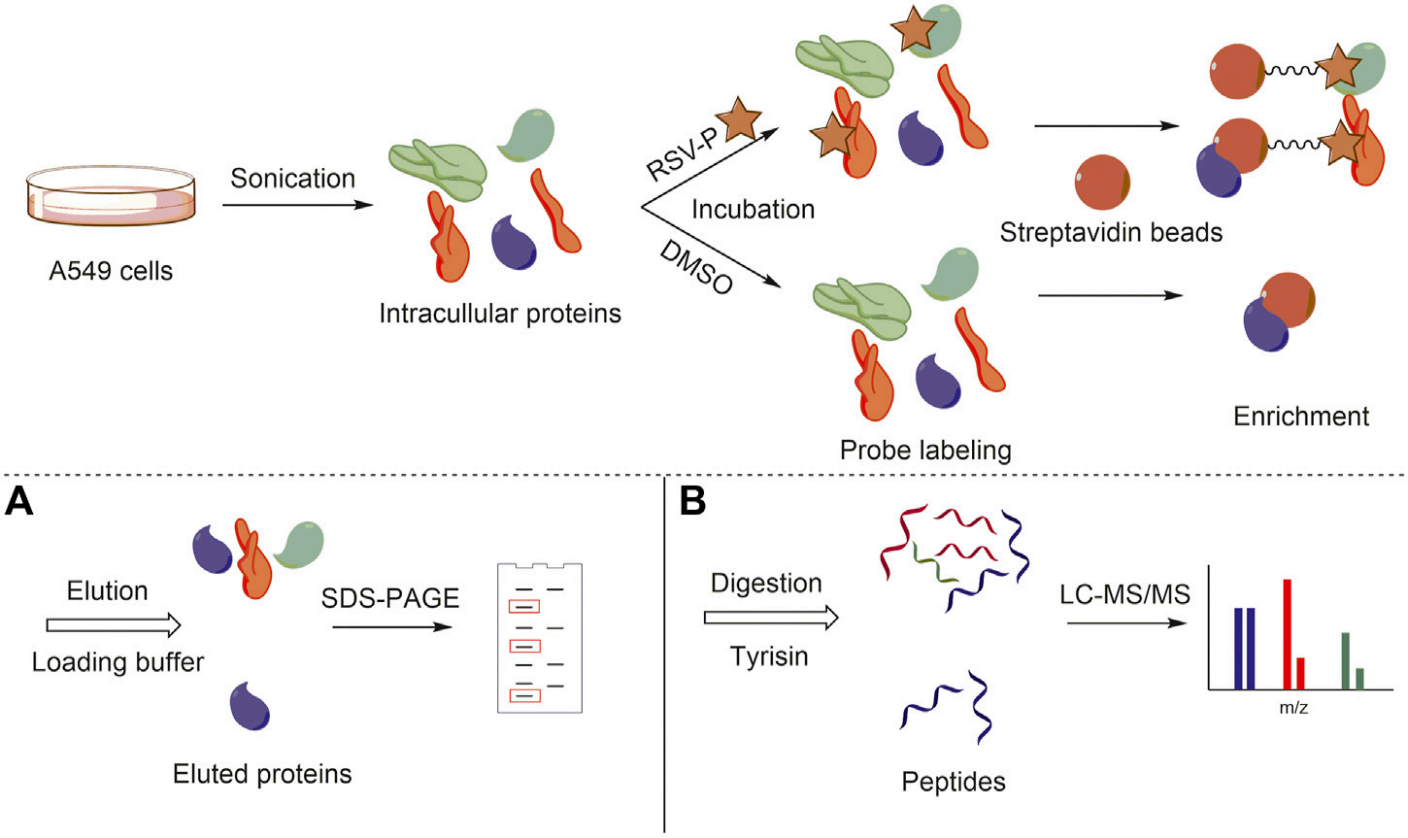
General workflow of chemical quantitative proteomic approach for target identification. **(A)** Target protein visualization with SDS-PAGE; **(B)** Target protein identification with LC-MS/MS.

**FIGURE 3:**
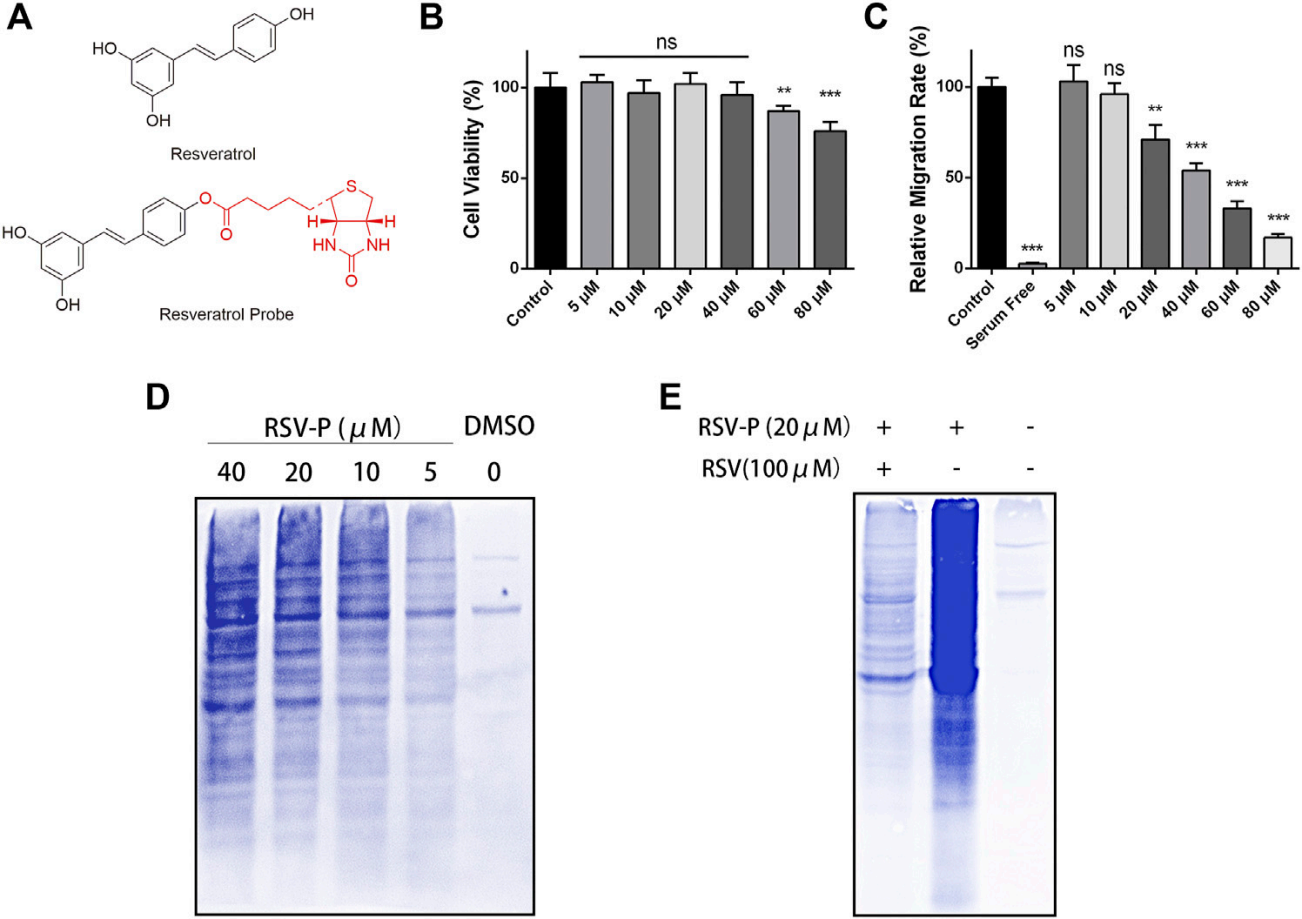
**(A)** Chemical structure of RSV and RSV-P; **(B)** Effect of different concentrations of RSV-P on A549 cell viability for 48 h; **(C)** Effect of different concentrations of RSV-P on A549 cell migration for 24 h; **(D)** The labeling proteins in A549 cells treated with different concentrations of RSV-P; **(E)** RSV competitively inhibits the interaction between RSV-P and target proteins. **p < 0.01, ***p < 0.005.

To elucidate the mechanism of action of RSV’s antimigration effect on A549 cells, a quantitative chemical proteomics approach was applied to identify the protein targets of RSV in A549 cells, as illustrated in Figure 2. Briefly, based on the structure-activity relationship of RSV (Chen et al., 2018), we first synthesized an RSV probe (RSV-P) derived from RSV (Figure 3A). The structure of RSV-P was validated with NMR and MS (Supplementary Figures S3, S4). In the structure of RSV-P, a biotin tag is linked to the 4′-hydroxy group in the RSV structure so that the probe can be easily enriched with streptavidin beads. To confirm that RSV-P retained similar pharmacological activity to RSV, we treated A549 cells with different concentrations of RSV-P for 24 or 48 h, and conducted CCK-8 and RTCA assays to examine the effect of RSV-P on A549 cell proliferation and migration. As shown in Figures 3B,C, RSV-P displayed no cytotoxicity to A549 cells at the concentration ranging from 0 to 40 μM but remarkably inhibited cell migration in a concentration-dependent manner (the IC_50_ value was 43.3 μM), indicating that RSV-P exerts similar activities to RSV. Next, the cultured A549 cells were lysed through sonication on ice to prevent the intracellular proteins from denaturing, and the lysates were incubated with RSV-P or with DMSO (control). After enrichment with streptavidin beads, the pulled-down proteins were digested with trypsin, and the obtained peptides were identified with liquid chromatography-mass spectrometry (LC-MS). To further confirm the specificity of the RSV-P pulled-down targets, we utilized different concentrations of RSV-P to treat A549 cell lysates, followed by streptavidin bead enrichment, protein elution, SDS-PAGE separation and Coomassie bright blue staining, and we found that an increasing pool of proteins was labeled with increasing concentrations of RSV-P (Figure 3D). Moreover, with the same method, it was observed that RSV could competitively inhibit the interaction between RSV-P and target proteins (Figure 3E), indicating that RSV-P covers the same target proteins as RSV.

### Targets Validation and Proteomic Analysis

**FIGURE 4:**
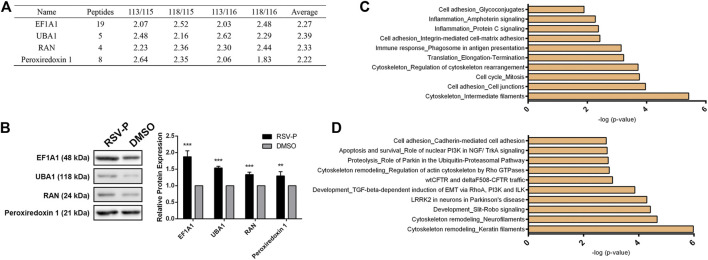
**(A)** Representative target proteins of RSV in A549 cells; **(B)** Western-blotting validation of the selected RSV’s target proteins; **(C)** RSV-related cellular processes from proteomic analysis; **(D)** Pathways regulated by RSV from proteomic analysis. **p < 0.01, ***p < 0.005.

iTRAQ coupled with MS/MS was employed to quantitatively identify the protein targets of RSV-P. Peptides from different groups were reacted with different iTRAQ reagents (RSV-P with 113 and 118; DMSO with 115 and 116). To ensure the specificity of the results, a strict cutoff threshold was employed: *p*-value <0.05 and average iTRAQ ratio >2, and 38 proteins were identified as the specific targets of RSV in A549 cells from a total of 1163 proteins (Supplementary Table S1). Four representative proteins from the target list, EF1A1, UBA1, RAN and Peroxiredoxin 1, were selected, and their interactions with RSV were further verified by immunoblotting (Figures 4A,B). Subsequently, we performed MetaCore analysis to predict RSV-modulated cellular processes and pathways according to the identified protein targets. RSV was involved in various cellular processes, including cell adhesion, inflammation, immune response, translation, cytoskeleton and cell cycle (Figure 4C). Among them, the cytoskeleton and cell adhesion are the top two RSV-related processes, which are directly correlated with cell migration. In addition, pathway analysis showed that RSV regulated several signaling pathways in A549 cells, among which cytoskeletal remodeling, cell adhesion and EMT were related to cell migration (Figure 4D). These results indicate that RSV affects A549 cell migration, which is consistent with our findings above.

### Resveratrol Inhibits A549 Cell Migration by Regulating Rho GTPase-Dependent Cytoskeletal Remodeling

**FIGURE 5:**
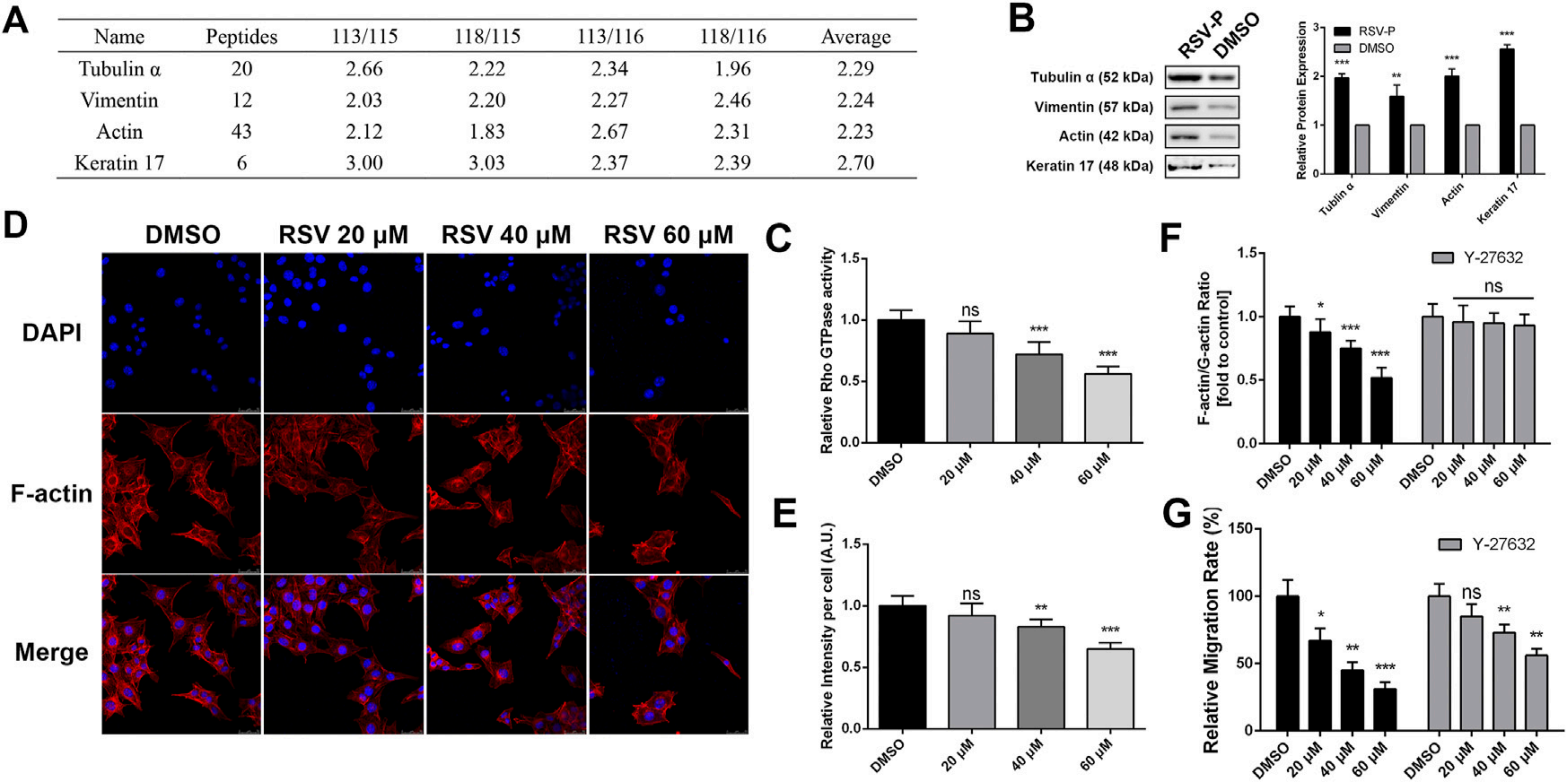
**(A)** The target proteins of RSV involved in cytoskeleton remodeling; **(B)** Western blotting validation of the cytoskeleton remodeling-related targets; **(C)** Activity of RhoA GTPase in A549 cells induced by different concentrations of RSV treatment; **(D)** Representative confocal images of A549 cells treated with different concentrations of RSV stained with rhodamine-phalloidin and DAPI; **(E)** Graph indicates the quantifications of mean fluorescence intensity of the confocal images of panels **(D)**; **(F)** The effects of different concentrations of RSV on the ratio of F-actin vs. G-actin expression in A549 cells with or without Y27632 pretreatment; **(G)** The effects of different concentrations of RSV on A549 cell migration with or without Y27632 pretreatment. *p < 0.05, **p < 0.01, ***p < 0.005.

Cytoskeletal remodeling is a complex process that leads to membrane edge extension and adhesive contact and stress fiber formation (Mclean et al., 2005), and it is closely linked to cell migration (Sayed et al., 2008; Du et al., 2017). Our target identification results showed that RSV directly bound to tubulin α, vimentin, actin and keratin 17 (Figure 5A), which are involved in cytoskeletal remodeling, and all of them were validated by western blotting (Figure 5B). In addition, pathway analysis indicated that RSV regulates Rho GTPase-dependent cytoskeletal remodeling. Considering the pharmacologic activity study of RSV, we hypothesized that RSV might inhibit A549 cell migration by regulating Rho GTPase-dependent cytoskeleton remodeling. Reports have shown that the Rho family protein RhoA is closely involved in cell migration, invasion and cytoskeletal assembly (Raftopoulou and Hall, 2004; Mattila and Lappalainen, 2008; Kardash et al., 2010); therefore, we first examined the effect of RSV on RhoA GTPase activity in A549 cells with a RhoA G-Lisa Kit. The results showed that RSV induced a significant decrease in RhoA GTPase activity in a concentration-dependent manner (Figure 5C). Treatment with RSV decreased actin polymerization in a concentration-dependent manner, as indicated by decreased qualitative (Figure 5D) and quantitative (Figure 5E) rhodamine-conjugated phalloidin fluorescence. To examine the connection between RhoA GTPase activity and actin remodeling, we pretreated A549 cells with Y-27632, an inhibitor of Rho-associated protein kinase (ROCK), a downstream effector of RhoA GTPase. The inhibition of actin polymerization by RSV in A549 cells, assessed by measuring the F- vs. G-actin ratio by western blotting, was attenuated by Y-27632 treatment for 1 h prior to RSV administration (Figure 5F), demonstrating that RSV regulates cytoskeletal remodeling through the Rho GTPase pathway. Furthermore, the effect of Y-27632 on RSV-mediated inhibition of A549 cell migration was also evaluated, and the results showed that Y-27632 partly rescued the inhibitory effect of RSV on cell migration (Figure 5G), revealing that RSV inhibits A549 cell migration by regulating Rho GTPase-dependent cytoskeletal remodeling.

### Resveratrol Suppresses TGFβ-Dependent EMT in A549 Cells to Inhibit Cell Migration.

Epithelial-to-mesenchymal transition (EMT) is defined as the process by which nonmotile, polarized epithelial cells lose their cell-cell junctions and convert into individual, motile mesenchymal cells, which is a key step in cancer cell migration, invasion and metastasis (Ha et al., 2014; Wang et al., 2017). Transforming growth factor β (TGFβ) is a major driving force of the EMT genetic program (Cufí et al., 2010). In our list of RSV targets, vimentin and actin were closely related to the EMT process, and our proteomics analysis indicated that RSV was involved in the TGFβ-dependent induction of EMT. Due to the relationship between EMT and cell migration, we inferred that RSV might affect EMT through the TGFβ pathway in A549 cells to inhibit cell migration, and a series of *in vitro* assays were performed to validate this hypothesis.

**FIGURE 6:**
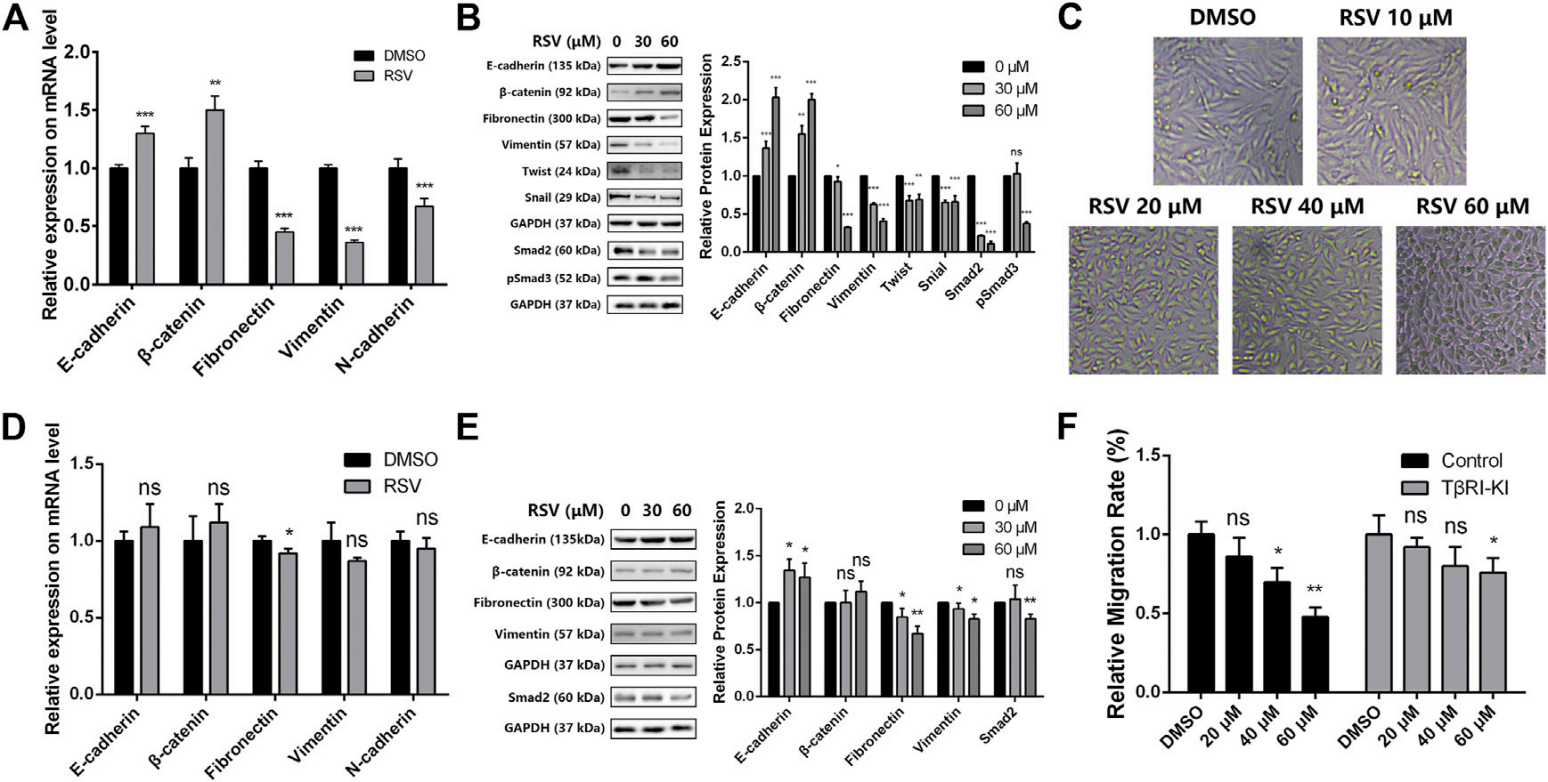
**(A)** Effect of RSV on EMT markers expression in A549 cells from qPCR assay; **(B)** Effect of RSV on EMT and TGFβ markers expression from western blotting; **(C)** A549 cell morphology with treatment of different concentration of RSV; **(D)** Effect of RSV on EMT markers expression in A549 cells pretreated with TβRI-KI from qPCR assay; **(E)** Effect of RSV on EMT and TGFβ markers expression in A549 cells pretreated with TβRI-KI from western blotting; **(F)** Effect of RSV on A549 cell migration with or without the pretreatment of TβRI-KI. *p < 0.05, **p < 0.01, ***p < 0.005.

It has been clearly established that Twist and Snail are regulators of EMT, and prevention of EMT causes an increase in epithelial marker proteins (E-cadherin and β-catenin) and a decrease in mesenchymal markers (fibronectin, vimentin, and N-cadherin) (Kim et al., 2012). In addition, Smad2 and pSmad3 are two biomarkers of TGFβ pathway. Therefore, we examined the effect of RSV on the expression of these EMT and TGFβ regulators and markers in A549 cells to confirm whether RSV affects TGFβ-dependent EMT. The results showed that RSV caused a striking upregulation of epithelial markers (E-cadherin and β-catenin) and a concomitant downregulation of EMT regulators (Twist and Snail), mesenchymal markers (Fibronectin, Vimentin and N-cadherin) and TGFβ pathway makers (Smad2 and pSmad3) at the mRNA and protein levels (Figures 6A,B). Moreover, a cell morphological study showed that A549 cells treated with RSV changed from a spindle-like mesenchymal phenotype to a round shape in a concentration-dependent manner (Figure 6C). These results reveal that RSV inhibits EMT in A549 cells to suppress migration.

Next, to confirm whether RSV regulates EMT in A549 cells through the TGFβ pathway, a TGFβ type I receptor kinase inhibitor (TβRI-KI) was applied (Bandyopadhyay et al., 2010). A549 cells were pretreated with TβRI-KI prior to RSV administration, and the expression of EMT and TGFβ markers in A549 cells was checked again with qPCR and western blotting. RSV-induced upregulation of epithelial markers and downregulation of mesenchymal and TGFβ markers were drastically reduced by TβRI-KI (Figures 6D,E), indicating that RSV regulates EMT in a TGFβ-dependent manner. Considering that EMT is closely related to cell migration, we also evaluated the effect of RSV on A549 cell migration with or without pretreatment with TβRI-KI (Figure 6F). TβRI-KI partly rescued the inhibitory effect of RSV on A549 cell migration. All the data demonstrate that RSV suppresses TGFβ-dependent EMT in A549 cells to inhibit cell migration.

### *In Vivo* Experiments Validated the Inhibitory Effect of Resveratrol on EMT in A549 Cells

**FIGURE 7:**
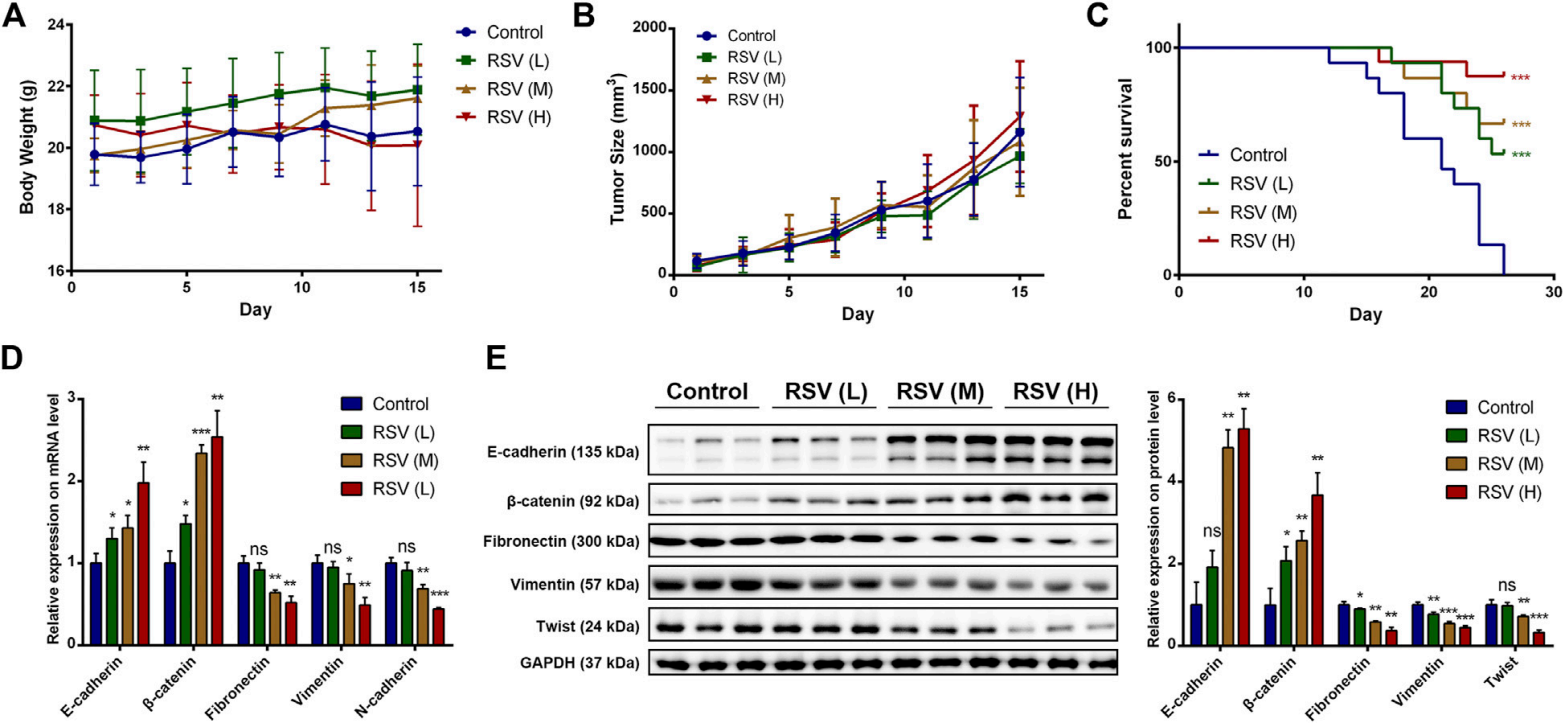
**(A)** Effect of RSV on body weight of model mice; **(B)** Effect of RSV on tumor size of model mice; **(C)** Effect of RSV on survival rate of model mice; **(D)** Effect of RSV on EMT markers expression in tumor from qPCR assay; **(E)** Effect of RSV on EMT markers expression in tumor from western blotting. *p < 0.05, **p < 0.01, ***p < 0.005.

Finally, we verified the inhibitory effect of RSV on EMT in A549 cells in an animal model. The tumor-bearing nude mice were randomly divided into four groups, which were intraperitoneally injected with 1% DMSO in PBS, a low concentration of RSV (15 mg/kg), a medium concentration of RSV (30 mg/kg) or a high concentration of RSV (50 mg/kg) each other day. The body weight and tumor size were measured. No significant difference in body weight of mice from the four groups was observed (Figure 7A), indicating the biological safety of RSV. The tumor size results showed that RSV did not exert an inhibitory effect on tumor size (Figure 7B), which was in line with our *in vitro* data. The tumor xenografts images and tumor weight at the study end point also confirmed the conclusion (Supplementary Figures S5, S6). However, RSV remarkably improved the survival rate of tumor-bearing mice in a concentration-dependent manner (Figure 7C). Therefore, we concluded that RSV exerts antitumor effects in A549 tumor-bearing mice not by inhibiting tumor growth. Finally, the mice were sacrificed when the tumors grew to about 1500 mm^3^, and the genomes and proteins were extracted from the tumors for subsequent experiments. We examined the expression of some key proteins with qPCR and western blotting. The qPCR results showed that RSV significantly increased the expression of E-cadherin and β-catenin and decreased the expression of fibronectin, vimentin and N-cadherin at the mRNA level in a concentration-dependent manner (Figure 7D), suggesting that RSV inhibited EMT in A549 cells *in vivo*. Consistently, western blotting results showed that RSV strikingly upregulated E-cadherin and β-catenin expression and downregulated fibronectin and vimentin expression at the protein level in a concentration-dependent manner (Figure 7E).

## Discussion

Resveratrol, a polyphenol present in a variety of fruits and vegetables, has been reported to prevent many diseases, such as cancer (Aida et al., 2015; Tan et al., 2016), cardiovascular disease (Zordoky et al., 2015), and Alzheimer’s disease (Turner et al., 2015). Of interest, RSV exerts anticancer activity in various cancer cell lines, including prostate cancer cells (Hsieh and Wu, 1999), breast cancer cells (Lu and Serrero, 1999), colonic cancer cells (Schneider et al., 2000), and ovarian cancer cells (Opipari et al., 2004). Notably, many studies have shown that RSV inhibits human A549 lung cells by affecting a variety of cellular processes, including the cell cycle (Yuan et al., 2015), apoptosis (Kim et al., 2003), autophagy (Hu et al., 2016), mitochondrial dysfunction (Gu et al., 2016), invasion and metastasis (Wang et al., 2013), depending on different experimental conditions and systems. Although researchers have investigated the mechanism of action (MOA) of RSV in A549 cells, its direct protein targets remain elusive, which increases the difficulty of elucidating the molecular mechanism and discovering new therapeutic targets. Moreover, MOA studies have shown that RSV is involved in many signaling pathways, such as the p21WAF1/CIP1, pRB, Bax, NF-κB (Kim et al., 2003), p53 (Yuan et al., 2015), Ca^2+^/AMPK/mTOR (Zhang et al., 2013), PKC-α, and NADPH oxidase (Hsu et al., 2018) signaling pathways, in A549 cells, but the RSV’s accurate MOA in A549 cells is not clear. Therefore, in the present study, we applied a quantitative chemical proteomics approach to identify the protein targets of RSV in A549 cells and elucidate the MOA of RSV based on these targets.

We first examined the effect of RSV on two human lung cancer cells, A549 and H226. Results showed that low concentration of RSV displayed no obvious cytotoxicity against A549 or H226 cells, but remarkably suppressed cell migration in a concentration- and time-dependent manner. The effect of RSV on cell viability was different from some of the previous studies. For example, Park et al. reported that 25 μM RSV exerted remarkable cytotoxicity against A549 cells after 48 h treatment (Kim et al., 2003). Similar results were also obtained by Zhang and Fan (Zhang et al., 2013; Fan et al., 2020). However, there are also some reports that support our conclusion. Yuan et al. showed that RSV displayed no cytotoxicity against A549 cells under the concentration of 50 μM after 24 or 48 h treatment (Yuan et al., 2015), which was also confirmed by another study (Wang et al., 2013). In addition, Kim et al. showed that RSV exhibited no inhibitory effect on A549 cell viability even at the concentration of 100 μM for 24 h treatment. Therefore, the toxic critical concentration of RSV against A549 cells remains controversial, but it does not influence the conclusion of the study, because RSV inhibits A549 cell migration at a very low concentration.

As small molecules and drugs always bind to their target proteins in cells to enhance or inhibit protein activity but do not affect their expression (Ollivier et al., 2003; Jost and Weissman, 2018), our proteomics approach of target identification guides us to a more comprehensive understanding of the MOA of RSV compared to the traditional proteomics approach that identifies only differentially expressed proteins. As an important branch of proteomics, quantitative chemical proteomics integrates diverse approaches in synthetic chemistry, cellular biology and mass spectrometry (Bantscheff et al., 2009). It is an approach to comprehensively fish and identify multiple protein targets of active small molecules in an unbiased manner and has been widely applied in target identification and MOA study of numerous active natural products, such as curcumin (Wang et al., 2016b), artemisinin (Wang et al., 2015) and zerumbone (Kalesh et al., 2015). Previous reports showed that the double bond within the structure of RSV plays an important role in RSV’s anti-migration activity (Chen et al., 2018), based on which we synthesized the molecular probe of RSV by connecting a biotin group to the 4′-hydroxy group in RSV for subsequent enrichment. Activity evaluation indicated that RSV-P retains the migration inhibitory effect of RSV, and the competing experiment showed that RSV-P competitively inhibits the binding of RSV to target proteins, revealing that RSV-P covers the same protein targets as RSV, and the targets identified by RSV-P that are involved in antimigration activity are confidently the RSV targets.

Cytoskeletal remodeling is an essential step for cell migration. Filippo Acconcia et al. reported that estrogen and tamoxifen induced cytoskeletal remodeling and migration in endometrial cancer cells (Acconcia et al., 2006). Loredana Pellegrino et al. showed that cytoskeleton modeling and cell motility were regulated by miR-23b synchronously by directly targeting multiple transcripts (Pellegrino et al., 2013). Therefore, regulating cytoskeletal remodeling has been a promising target to suppress cancer cell migration (Wang et al., 2019). Here, our proteomic data revealed the relationship between RSV and cytoskeletal remodeling in A549 cells, which was validated with subsequent assays. As a member of the Rho family that is closely involved in regulating cell migration, invasion and cytoskeletal assembly (Raftopoulou and Hall, 2004; Mattila and Lappalainen, 2008; Kardash et al., 2010), RhoA was found to be inhibited by RSV in A549 cells in a concentration-dependent manner, indicating that RSV might regulate cytoskeletal remodeling through the RhoA pathway. To confirm this hypothesis, we employed an inhibitor of ROCK, Y-27632, to block the RhoA pathway. The results showed that Y-27632 attenuated the effect of RSV on cytoskeletal remodeling and migration of A549 cells to some extent, suggesting that RSV regulates A549 cell cytoskeletal remodeling through the RhoA pathway and further inhibits A549 cell migration partly by regulating cytoskeletal remodeling.

Epithelial-to-mesenchymal transition is a key developmental process that cancer cells hijack to increase their aggressiveness and invasive potential (Nieto, 2011). The induction of EMT is directly relevant to cell migration activation, and suppressed EMT always leads to cell migration inhibition. The effect of RSV on EMT has been broadly investigated in a variety of cancer cells, such as pancreatic cancer cells (Li et al., 2013) and lung cancer cells (Wang et al., 2013). Of interest, Wang et al. reported that RSV inhibited TGF-β1-induced EMT and suppressed cell invasion and metastasis in A549 cells (Wang et al., 2013), which is in line with our findings. In this study, we further identified the protein targets of RSV that are related to its EMT inhibition effect, including vimentin and actin. Moreover, we utilized a TGFβ type I receptor kinase inhibitor (TβRI-KI) to examine whether RSV inhibited EMT through the TGFβ pathway and suppressed A549 cell migration by inhibiting EMT. TβRI-KI partly rescued the inhibitory effect of RSV on EMT and cell migration in A549 cells, demonstrating that RSV suppresses A549 cell migration partly by inhibiting the EMT pathway. Our *in vivo* experiment also confirmed this conclusion. The results showed that RSV displayed no inhibitory effect on the tumor size of the model mice but significantly improved the survival rate, which demonstrated that RSV exerts its antitumor activity not through inhibiting tumor growth but through other approaches. Subsequent qPCR and western blotting showed that RSV significantly inhibited EMT pathways in tumor tissues, indicating that RSV suppresses EMT to inhibit tumor cell migration, thereby exerting antitumor activity.

The first death of the negative control group occurred on the day 12, and the tumor volume of the mouse was about 680 mm^3^. Although the mice possess simplex genetic background, there exists individual difference. The direct reason of the death might not be the tumor, but it did happen. Nevertheless, in order to guarantee the objectivity of the results, the case was recorded and reflected in our results. The second and subsequent deaths occurred on the day 15 or later, and the tumor volumes of these mice were larger than 1500 mm^3^. Therefore, we believe that these deaths could be attributed to the tumor according to previous reports (Shi et al., 2016; Yang et al., 2018).

Based on our findings, we could reason that RSV inhibits tumor metastasis *in vivo*. Corroborating the hypothesis, the effect of RSV on tumor metastasis *in vivo* has been examined in various cancer cell lines by previous studies. For example, our group have reported that RSV suppressed B16F10 melanoma cells migration *in vivo* through an epigenetic pathway, but not influenced tumor growth (Chen et al., 2018). Qin et al. showed that RSV inhibited the lung metastases of LoVo human colon cancer cells *in vivo* (Ji et al., 2015). Of interest, Lina et al. showed that RSV regulated SIRT1 pathway to inhibit A549 *in vivo* metastasis (Sun et al., 2013). Liwei et al. reported that RSV suppressed A549 tumor metastasis by inhibiting platelet-mediated angiogenic responses (He et al., 2017). Therefore, we chose a xenograft mouse model but not a passive transfer model as our experiment system.

Although our results indicate that RSV separately regulates RhoA-dependent cytoskeletal remodeling and TGFβ-dependent EMT to inhibit A549 cell migration, many reports have shown RhoA pathway is functionally linked to EMT in a variety of cancer cell lines (Korol et al., 2016; Salvi and Thanabalu, 2017). It has been well established that RhoA is the upstream pathway of EMT (Bhowmick et al., 2001; Tavares et al., 2006). For example, Shuai Zhu et al. have reported that ASIC1 and ASIC3 contribute to acidity-induced EMT of pancreatic cancer through activating Ca^2+^/RhoA pathway (Zhu et al., 2017). Lin et al. have shown that FPPS mediates TGF-β1-induced non-small cell lung cancer cell invasion and the EMT process via the RhoA/Rock1 pathway (Lin et al., 2018). However, whether RSV inhibits EMT through RhoA pathway has not been reported. Our study has elucidated the role of RSV in RhoA pathway and EMT in A549 cells, but their relationship remains elusive, which could be further investigated in the future.

Based on a cell-based study, we discovered that low concentration of RSV exhibits no cytotoxicity in A549 cells but significantly inhibits cell migration. To elucidate the mechanism involved, we adopted a chemical quantitative proteomics approach to identify the protein targets of RSV in A549 cells. A probe derived from RSV was synthesized to identify the targets, and iTRAQ coupled with LC-MS/MS was employed to identify the targets. As a result, 38 out of 1163 proteins were identified as confident targets of RSV in A549 cells. With Metacore analysis, we found that the targets of RSV were involved in various cellular processes and pathways, among which cytoskeletal remodeling and EMT were related to cell migration. Subsequent *in vitro* assays confirmed that RSV regulated cytoskeletal remodeling through the RhoA pathway and inhibited EMT through the TGFβ pathway, which contributed to RSV’s antimigration activity in A549 cells. The *in vivo* experiments also supported this conclusion. To summarize, RSV inhibits A549 cell migration by binding multiple targets to regulate cytoskeletal remodeling and inhibit EMT in A549 cells.

Therefore, our study reinforces the anticancer activity of RSV and elucidates the mechanism of RSV’s antimigration effect on A549 cancer cells with a chemical quantitative proteomics approach, providing not only evidence that RSV can be further developed as a potential anticancer agent but also future insights for the nutraceutical development of RSV.

## Data Availability

The datasets presented in this study can be found in online repositories. The names of the repository/repositories and accession number(s) can be found in the article/Supplementary Material.
