# Supplementary figures and images for: Quantitative Chemical Proteomics Reveals Resveratrol Inhibition of A549 Cell Migration Through Binding Multiple Targets to Regulate Cytoskeletal Remodeling and Suppress EMT
Source: Front Pharmacol. 2021 Mar 26;12:636213. doi: 10.3389/fphar.2021.636213 (PMC8044895; doi:10.3389/fphar.2021.636213)

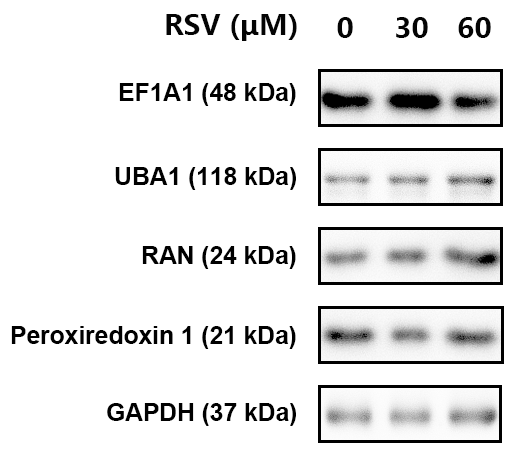

Supplement: Supplementary file 1 [file image1.tif]
