# Supplementary material for: Quantitative Chemical Proteomics Reveals Resveratrol Inhibition of A549 Cell Migration Through Binding Multiple Targets to Regulate Cytoskeletal Remodeling and Suppress EMT
Source: Front Pharmacol. 2021 Mar 26;12:636213. doi: 10.3389/fphar.2021.636213 (PMC8044895; doi:10.3389/fphar.2021.636213)
Supplement: Supplementary file 2 [file datasheet1.docx]

Supplementary Material

## Supplementary Figures


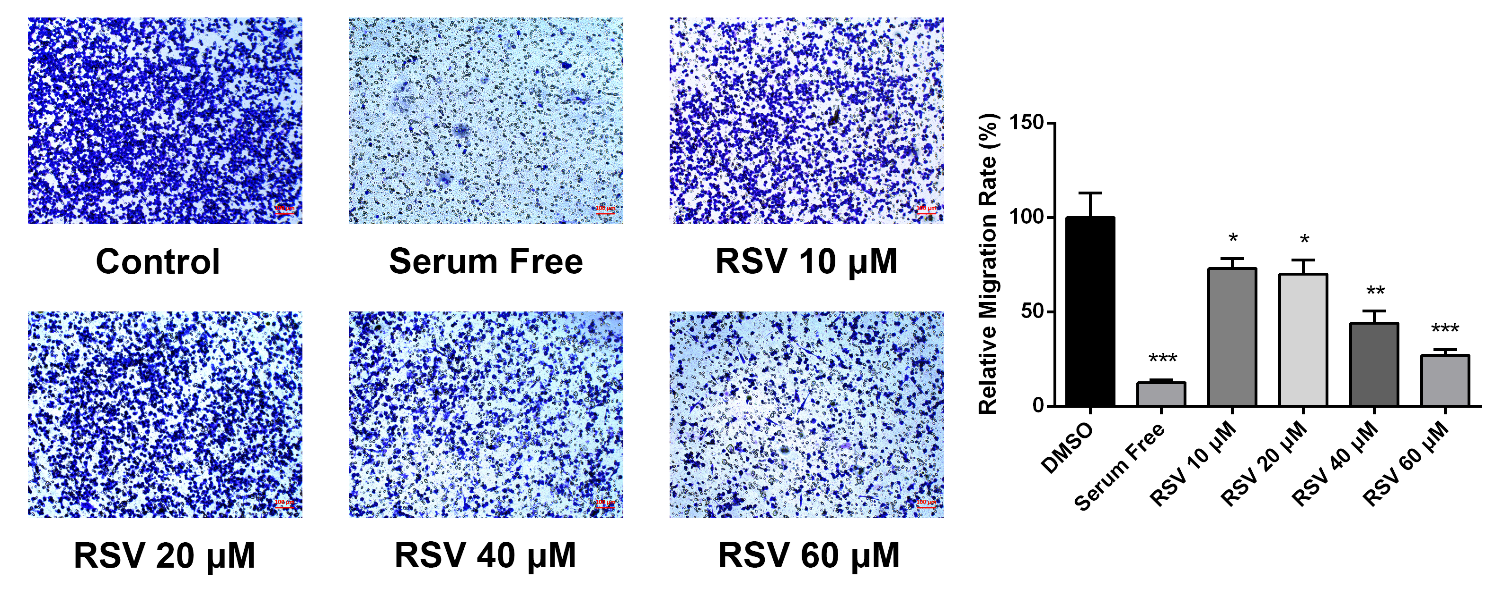
**Supplementary Figure 1.** The effect of different concentrations of RSV on A549 cell migration from trans-well assay.


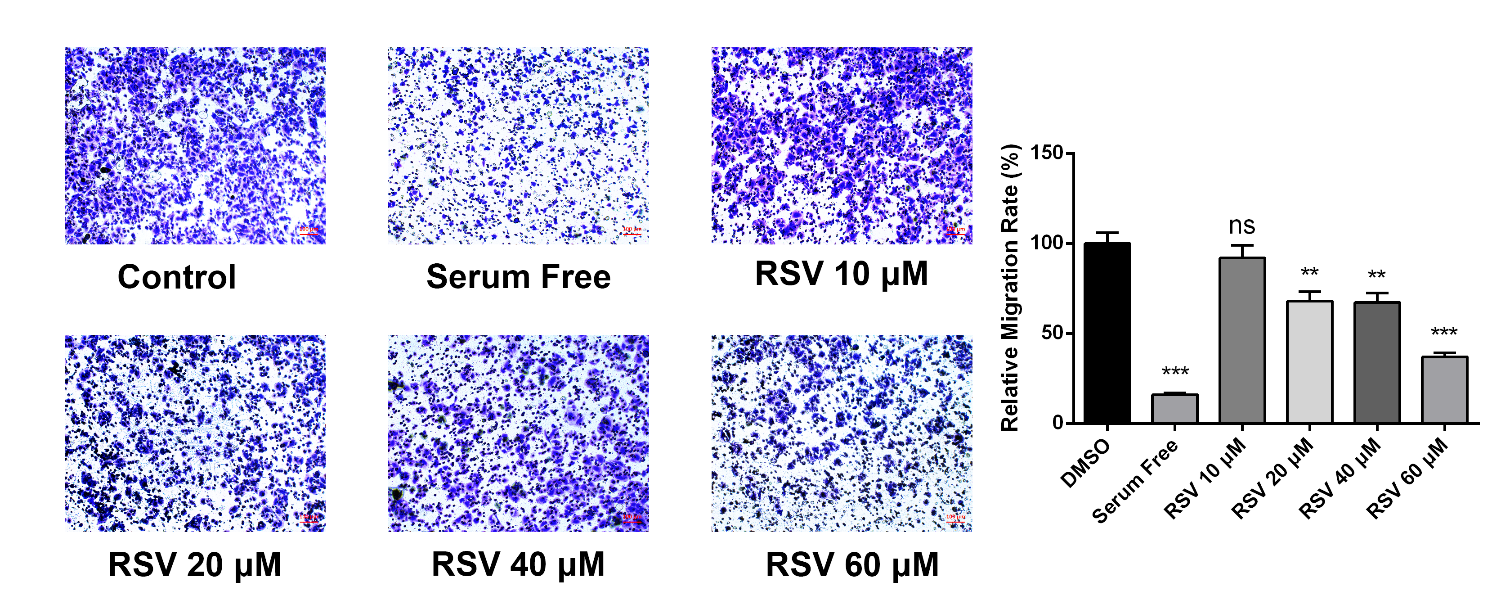


**Supplementary Figure 2.** The effect of different concentrations of RSV on H226 cell migration from trans-well assay.


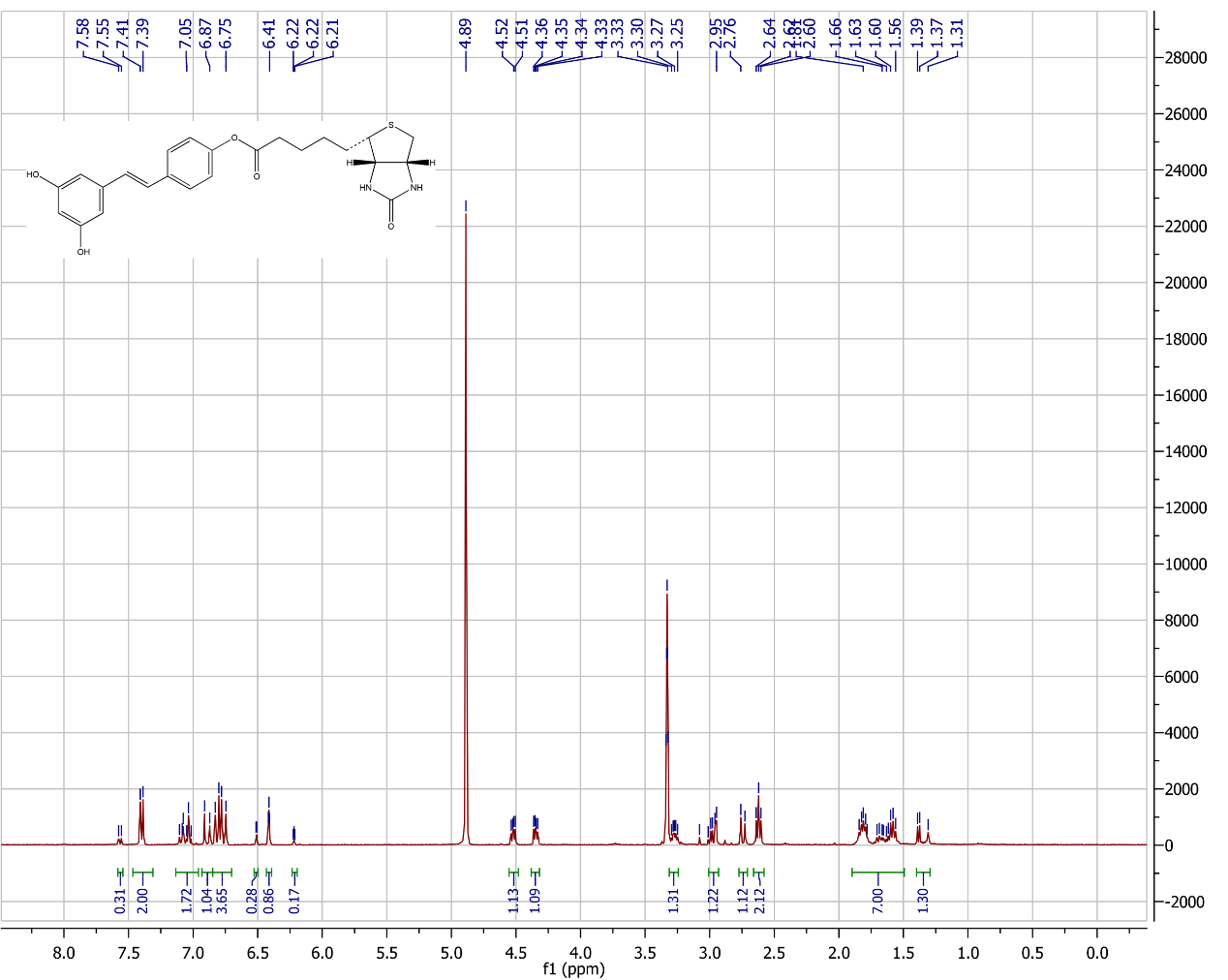


**Supplementary Figure 3.** ^1^H-NMR of RSV-P.


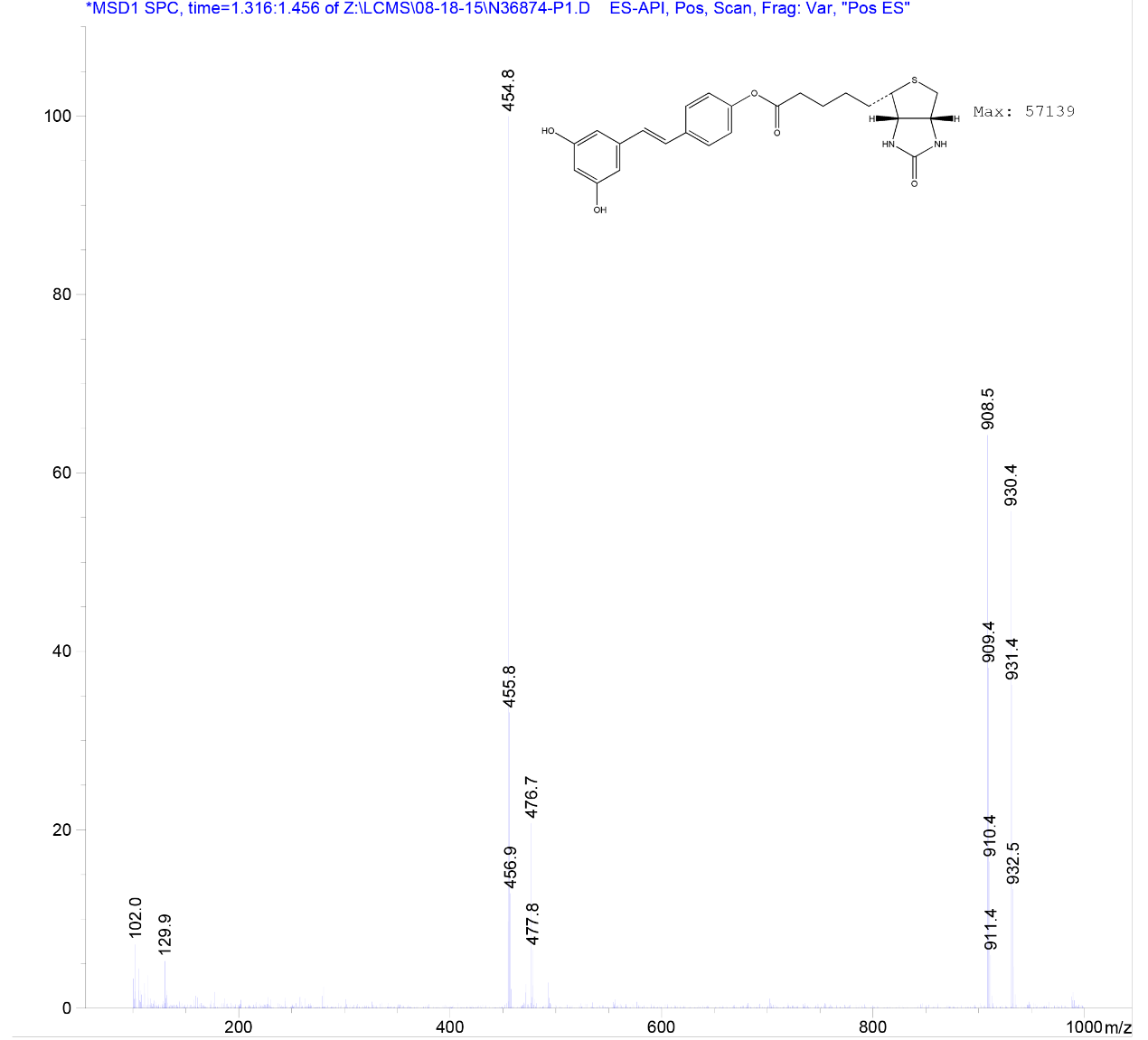


**Supplementary Figure 4.** Mass spectrum of RSV-P


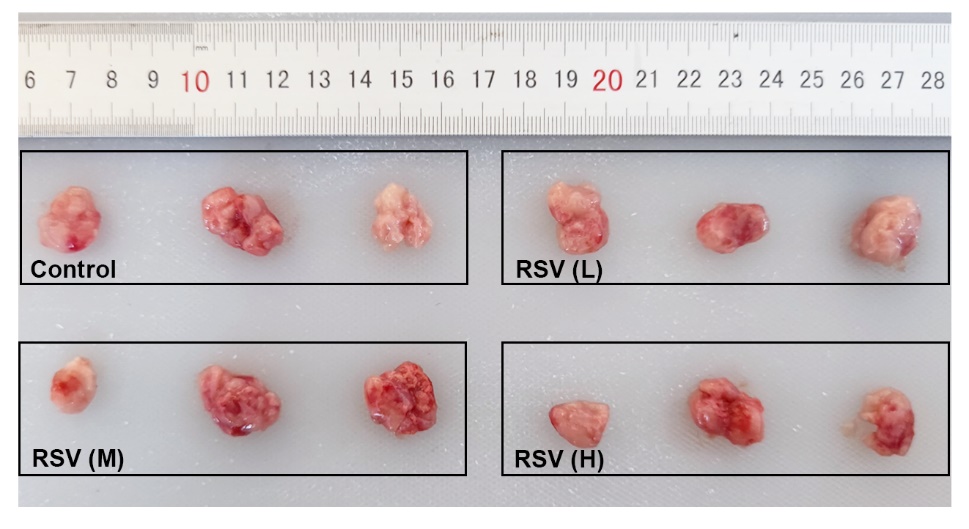


**Supplementary Figure 5.** Tumor xenografts images at the sutdy end point





**Supplementary Figure 6.** Tumor weight at the sutdy end point

## Supplementary Tables

**Supplementary Table 1.** List of RSV target proteins identified with chemical proteomics approach in A549 cells.

| Swiss-Prot ID | Name | Species | Peptides(95%) | 113/115 | 118/115 | 113/116 | 118/116 | average |
| --- | --- | --- | --- | --- | --- | --- | --- | --- |
| P50990 | T-complex protein 1 subunit theta | HUMAN | 2 | 2.3177 | 2.7699 | 2.9480 | 3.5232 | 2.8897 |
| Q13547 | Histone deacetylase 1 | HUMAN | 15 | 2.8783 | 3.2002 | 2.3906 | 2.7280 | 2.7993 |
| P13639 | Elongation factor 2 | HUMAN | 16 | 2.5923 | 2.6685 | 2.7615 | 2.8426 | 2.7162 |
| P27348 | 14-3-3 protein theta (Fragment) | HUMAN | 2 | 2.4714 | 2.6647 | 2.7230 | 2.9360 | 2.6988 |
| Q04695 | Keratin, type I cytoskeletal 17 | HUMAN | 6 | 3.0034 | 3.0289 | 2.3697 | 2.3897 | 2.6979 |
| P36776 | Lon protease homolog, mitochondrial | HUMAN | 3 | 2.6878 | 3.3608 | 2.0890 | 2.6120 | 2.6874 |
| Q15393 | Splicing factor 3B subunit 3 | HUMAN | 3 | 2.7332 | 2.9208 | 2.3130 | 2.4717 | 2.6097 |
| P13667 | Protein disulfide-isomerase A4 | HUMAN | 2 | 2.1617 | 2.7301 | 2.3867 | 3.0143 | 2.5732 |
| P30101 | Protein disulfide-isomerase A3 | HUMAN | 6 | 2.1662 | 2.5262 | 2.4623 | 2.8714 | 2.5065 |
| P27708 | Carbamoyl-phosphate synthetase 2, aspartate transcarbamylase, and dihydroorotase | HUMAN | 2 | 2.4861 | 2.4234 | 2.5714 | 2.5066 | 2.4969 |
| Q15029 | Elongation factor Tu | HUMAN | 6 | 2.2167 | 2.0339 | 2.7707 | 2.5422 | 2.3909 |
| P22314 | Ubiquitin-like modifier-activating enzyme 1 | HUMAN | 5 | 2.4774 | 2.1611 | 2.6224 | 2.2876 | 2.3871 |
| P00558 | Phosphoglycerate kinase 1 | HUMAN | 6 | 1.9972 | 2.5205 | 2.1969 | 2.7725 | 2.3718 |
| P23921 | Ribonucleoside-diphosphate reductase large subunit | HUMAN | 2 | 2.5266 | 2.6485 | 2.0938 | 2.1947 | 2.3659 |
| P04075 | Fructose-bisphosphate aldolase | HUMAN | 3 | 2.6064 | 2.6856 | 2.0071 | 2.0681 | 2.3418 |
| P17844 | DEAD (Asp-Glu-Ala-Asp) box polypeptide 5 | HUMAN | 3 | 2.0993 | 2.1940 | 2.4799 | 2.5917 | 2.3412 |
| P62826 | GTP-binding nuclear protein Ran | HUMAN | 4 | 2.2253 | 2.3585 | 2.2991 | 2.4366 | 2.3299 |
| P23528 | Cofilin-1 | HUMAN | 3 | 2.5405 | 2.5106 | 2.1260 | 2.1010 | 2.3195 |
| Q9BQE3 | Tubulin alpha-1C chain | HUMAN | 20 | 2.6580 | 2.2237 | 2.3371 | 1.9552 | 2.2935 |
| P68104 | Elongation factor 1-alpha 1 | HUMAN | 19 | 2.0676 | 2.5234 | 2.0296 | 2.4770 | 2.2744 |
| P23396 | Ribosomal protein S3 | HUMAN | 6 | 2.0775 | 2.4145 | 2.0973 | 2.4375 | 2.2567 |
| P08670 | Vimentin | HUMAN | 12 | 2.0349 | 2.2032 | 2.2692 | 2.4568 | 2.2410 |
| P60709 | Actin beta | HUMAN | 43 | 2.1167 | 1.8305 | 2.6657 | 2.3052 | 2.2295 |
| Q06830 | Peroxiredoxin-1 (Fragment) | HUMAN | 8 | 2.6434 | 2.3460 | 2.0574 | 1.8259 | 2.2182 |
| Q13263 | Tripartite motif containing 28 | HUMAN | 3 | 1.9495 | 2.3205 | 2.0813 | 2.4773 | 2.2071 |
| Q6UB35 | Methylenetetrahydrofolate dehydrogenase (NADP+ dependent) 1 like pseudogene | HUMAN | 7 | 2.3896 | 2.5572 | 1.8606 | 1.9912 | 2.1996 |
| O43175 | Phosphoglycerate dehydrogenase | HUMAN | 7 | 2.0036 | 2.1288 | 2.1845 | 2.3210 | 2.1595 |
| P25205 | Minichromosome maintenance complex component 3 | HUMAN | 3 | 2.1047 | 1.9644 | 2.3582 | 2.2010 | 2.1571 |
| Q00610 | Clathrin heavy chain | HUMAN | 6 | 1.9771 | 2.3770 | 1.8896 | 2.2718 | 2.1289 |
| P19338 | Nucleolin | HUMAN | 3 | 1.8312 | 2.2544 | 1.9839 | 2.4424 | 2.1280 |
| Q96CM8 | Acyl-CoA synthetase family member 2, mitochondrial | HUMAN | 2 | 2.1451 | 2.5910 | 1.6971 | 2.0499 | 2.1208 |
| P60228 | Eukaryotic translation initiation factor 3 subunit E | HUMAN | 2 | 2.0816 | 1.9613 | 2.2754 | 2.1438 | 2.1155 |
| P35998 | Proteasome 26S subunit, ATPase 2 | HUMAN | 3 | 2.1607 | 2.3603 | 1.8670 | 2.0395 | 2.1069 |
| Q9Y6K1 | DNA (cytosine-5)-methyltransferase 3A | HUMAN | 7 | 2.1189 | 2.4339 | 1.7290 | 2.1159 | 2.0994 |
| O95373 | Importin-7 | HUMAN | 2 | 2.4051 | 2.0226 | 2.1002 | 1.7662 | 2.0735 |
| P49588 | Alanyl-tRNA synthetase 1 | HUMAN | 3 | 1.8350 | 2.1029 | 1.9977 | 2.2894 | 2.0562 |
| Q00839 | Heterogeneous nuclear ribonucleoprotein U | HUMAN | 9 | 2.0660 | 1.7014 | 2.4144 | 1.9883 | 2.0425 |
| P53396 | ATP citrate lyase | HUMAN | 6 | 2.0856 | 1.8188 | 2.1944 | 1.9136 | 2.0031 |
